# Nest preference and laying duration traits to select against floor eggs in laying hens

**DOI:** 10.1186/s12711-023-00780-8

**Published:** 2023-01-25

**Authors:** Lorry Bécot, Nicolas Bédère, Jenna Coton, Thierry Burlot, Pascale Le Roy

**Affiliations:** 1 PEGASE, INRAE, Institut Agro, 35590 Saint Gilles, France; 2 NOVOGEN, 22960 Plédran, France

## Abstract

**Background:**

Floor eggs, which are defined as eggs that hens lay off-nest, are a major issue in cage-free layer poultry systems. They create additional work for farmers because they must be collected by hand. They are also usually soiled or broken, which results in economic losses. Nonetheless, knowledge about the genetics of nesting behavior is limited. The aim of this study was to estimate genetic parameters for traits related to nest preference for laying and to time spent in the nests used for laying (laying duration).

**Methods:**

Two pure lines of laying hens were studied: 927 Rhode Island Red and 980 White Leghorn. Electronic nests were used to record the nesting behavior of these hens in floor pens from 24 to 64 weeks of age. Nest preference was studied based on the mean distance between nests used for laying and the percentage of nests used for laying. Laying duration was studied based on mean laying duration, mean duration in the nest before laying, and mean duration in the nest after laying. Genetic parameters were estimated for each line using a restricted maximum-likelihood method applied to a pedigree-based multi-trait animal model.

**Results:**

Estimates of genetic parameters were similar for the two lines. Estimates of heritability ranged from 0.18 to 0.37 for nest preference traits and from 0.54 to 0.70 for laying duration traits. Estimates of genetic correlations of these traits with clutch number or mean oviposition time were favorable. Positive genetic correlations were estimated between nest preference and laying rate in the nests or nest acceptance for laying (+ 0.06 to + 0.37).

**Conclusions:**

These results show that genetics influences traits related to nest preference and laying duration. Selecting hens that have no preference for particular nests and spend little time laying in the nests could help optimize nest use, reduce their occupation rate, and thus decrease the incidence of floor eggs in cage-free systems. Genetic correlations of these traits with other traits of interest related to hen welfare and egg quality have yet to be estimated.

**Supplementary Information:**

The online version contains supplementary material available at 10.1186/s12711-023-00780-8.

## Background

Most egg-production systems in the European Union are cage-free systems (55% of laying hens in 2021 [1]), such as barn, free-range, and organic systems. These systems have nests that enable laying hens to express natural behaviors such as nesting [2]. The nests also enable automated egg collection of eggs and maintain egg quality. Floor eggs, which are defined as eggs that hens lay off-nest, are a major issue in cage-free systems for several reasons. Collecting floor eggs by hand is laborious and time-consuming. Floor eggs cause economic losses because they are often soiled with droppings, broken, or eaten by the hens. Floor eggs also have more bacteria on the eggshell [3] and lower fertility and hatchability than eggs laid in the nests [4]. Hens that lay floor eggs display more nest-seeking behavior, less nest-building behavior, and less sitting before oviposition than hens that lay in the nests, which may indicate frustration and reduced welfare [5].

The incidence of floor eggs could be reduced by controlling environmental factors such as providing shaded and sheltered spaces, which hens prefer for laying [6]; lighting regime [7] and intensity [8]; enrichment with perches [9]; the feeding schedule [10]; and nest design [6, 11]. The tendency to lay floor eggs is a learned behavior, which emphasizes the importance of good environmental management and training of young hens [12]. Other studies have observed some hens showing a predisposition to lay floor eggs [5, 13, 14]. The percentage of floor eggs was found to be moderately to highly heritable, with estimates in the literature ranging from 0.39 to 0.52 [12, 15]. In addition to good environmental management, the presence of a genetic component for percentage of floor eggs may enable selection against laying of floor eggs. However, these studies were based on hens that were raised in small groups (12–18 individuals) by sire family, while hens in cage-free systems are raised in large groups and come from multiple families. To identify hens that lay floor eggs, a recent study suggested the use of genomic information from embryonic DNA, but also pointed out its limitations for application, such as the cost and labor associated with obtaining the DNA and the fact that only fertile eggs can be tested [12]. Currently, no technology exists to easily identify which hens lay floor eggs in large groups. To select hens against laying of floor eggs and to optimize nest use, some studies have suggested selecting for nesting behavior traits and using radio-frequency identification with electronic nests to record these traits at the individual level and in large groups [16–19].

Behavior traits related to nest preference and laying duration (i.e., the time spent in the nest used for laying) may be associated with laying of floor eggs. The choice of nest site is an important step in pre-laying behavior [2]. Some hens prefer to lay in nests that are already occupied by other hens, even when empty nests are available. This behavior, called gregarious nesting, results in welfare issues, with the risk of suffocation in the nest and production problems, with an increase in laying of floor eggs when the nest chosen for laying is full [20, 21]. One study classified hens based on their choice of nests (whether occupied or not) as gregarious, solitary, or intermediary nesters [22]. Hens may also prefer nests that are located in the corners of the pen, which implies non-optimal nest use [20, 21, 23, 24]. Selecting laying hens with no preference for particular nests could reduce gregarious nesting, optimize nest use, and thus reduce laying of floor eggs. Similarly, a higher rate of nest occupancy increases laying of floor eggs [25]. Hens may compete for access to the nests, thus forcing some hens to lay floor eggs [26]. Reducing the laying duration could also help reduce the occupancy rate in the nests, and thus reduce laying of floor eggs. However, to our knowledge, genetic parameters for nest preference traits for laying have not been estimated, and estimates of heritability of laying duration vary depending on the laying period and flock [17]. Relationships between these traits and other nest-related traits of interest, such as laying rate in nests and nest acceptance, are also not known.

Continuous records from electronic nests can help determine traits related to nest preference and laying duration for hens that are raised in large groups in cage-free systems. The present study aimed at describing phenotypes and estimating genetic parameters for traits related to nest preference and laying duration, as well as genetic correlations of these traits with other nest-related traits. It also explored changes in the genetics of laying duration traits with age.

## Methods

### Hens

The two pure lines used in this study were selected by the Novogen breeding company (Plédran, France). The total population consisted of two batches of a Rhode Island Red (RIR) line and two batches of a White Leghorn (WL) line (Table 1). All hens were raised on the floor until 17 weeks of age, and were then transferred to another barn adapted for egg production, where they stayed until 64 weeks of age in floor pens that contained one batch of one line. Each floor pen also housed approximately one rooster for every 10 hens for commercial breeding. In total, 927 RIR hens (from 98 sires and 348 dams) and 980 WL hens (from 99 sires and 352 dams) were phenotyped. The genetic structure was the same for the two populations, with about ten hens per sire and three hens per dam.

**Table 1:** Characteristics of the lines and batches of hens studied

Characteristic	Rhode Island Red		White Leghorn	
Batch year	2018–2019	2019–2020	2018–2019	2019–2020
Hens housed	455	552	513	598
Hens analyzed	403	524	509	471
Number of nests in the pen	100	120	80	120
Recording period (weeks of age)	24–58	24–64	24–64	24–64
Nest visits with oviposition	73,063	118,787	89,786	122,635
Floor eggs (%)	4.6	2.9	8.9	0.8

### Data acquisition

Data were collected using individual electronic nests developed by Novogen. Floor pens were equipped with 80–120 electronic nests (Table 1) divided into two rows (top and bottom). Radio-frequency identification was used to identify individual hens when they visited the nests, based on a transponder attached to one leg of each hen. The electronic nests identified the individual hen, the location of the nest, and the times of entry into and exit from the nest. Oviposition time was recorded using an egg sensor located behind each nest.

The hens gradually adapted to using the nests from 17 to 23 weeks of age. Data were collected daily for 40 weeks, when the hens were 24–64 weeks of age, except for the 2018–2019 batch of RIR, for which data collection was terminated at 58 weeks of age due to an infection (Table 1). The time of entry for nest visits with oviposition was used to calculate the clutch number (CN) and mean oviposition time (MOT) because it was strongly correlated with, and more reliable than, oviposition time [18], since eggs sometimes remained stuck in a nest until the hen left the nest, or even later (4.3% and 11.2% of eggs for RIR and WL, respectively). To compare the phenotypes to those reported in the literature, MOT was also calculated using oviposition time, when it was accurately measured (Table 2).

**Table 2:** Summary statistics for nest-related traits for Rhode Island Red and White Leghorn hens

Trait	Rhode Island Red					White Leghorn				
	n	Mean	SD	Min.	Max.	n	Mean	SD	Min.	Max.
LRN (%)	788	91.80	8.32	51.45	99.65	879	86.53	9.37	50.71	98.58
CN	788	3.98	3.88	1.00	32.00	879	8.26	5.92	1.00	40.00
MOT (hh:mm)	831	02:28	01:07	00:04	06:17	920	03:39	01:01	00:28	06:38
MDN (nests)	827	7.80	3.03	0.47	20.26	918	9.21	3.06	2.54	21.46
PNL (%)	802	43	13	9	81	895	59	16	16	96
MLD (min)	832	41	17	11	106	921	64	23	16	142
MDB (min)	826	27	10	8	66	920	30	11	10	75
MDA (min)	826	14	13	1	67	920	34	19	3	103

	n	0	1			n	0	1		
NAL (%)	927	14.99	85.01			980	10.31	89.69		

The percentage of the observed phenotypic variance explained by the fixed effect of the hen’s hatch date was ≤ 10%, except for MOT (13%) and MDN (22%) in White Leghorn

*LRN* laying rate in the nests, *CN* clutch number, *MOT* mean oviposition time, *MDN* mean distance between nests used for laying, *PNL* percentage of nests used for laying, *MLD* mean laying duration, *MDB* mean duration in the nest before laying, *MDA* mean duration in the nest after laying, *NAL* nest acceptance for laying (0 for hens with LRN < 50%, 1 otherwise), *n* number of hens with phenotype, *SD* standard deviation, *Min.* minimum, *Max.* maximum values

In this study, visits that did not lead to oviposition were not considered, which accounted for 26 and 36% of nests visits for RIR and WL, respectively. Indeed, only a few hens were responsible for these visits (i.e., 5 and 8% of hens for RIR and WL, respectively). The reasons for these visits could include testing several nests before laying, disturbance while laying, or using the nests as a refuge, but are difficult to identify. Most of the visits without oviposition occurred after the laying period (58% for both lines).

### Traits

Traits were calculated using the electronic nest records during the 40-week data collection period. Only hens that survived for at least half of the period were analyzed (Table 1). Egg production was defined as the laying rate in the nests (LRN):

1 $${\text{LRN}}=\frac{\text{Number of eggs laid in the nests} }{\text{Number of days alive during the recording period}} \times 100.$$

LRN, CN, MOT, and nest acceptance for laying (NAL) were calculated as described by [18]. In short, LRN phenotypes were kept for hens with LRN ≥ 50%, since low LRN can be caused by a variety of factors (e.g., preference for laying floor eggs, molting, repeated pauses) that make interpreting LRN more difficult. Phenotypes for CN were calculated as the number of pauses + 1. When a hen did not lay in a nest for a period of 1 to 4 days, she was either pausing or laying floor eggs. In such cases, the difference between the 24-h time on the day of the last nest visit before such a period and the 24-h time on the day of the first visit after the period was calculated (i.e., time difference between two times of entry for nest visits with oviposition, ignoring the difference in days). A time difference that decreased by more than 3 h for RIR or 3 h and 15 min for WL meant a reset of the biological clock to earlier morning hours and, therefore, that the hen had done at least one day of pause. Conversely, a time difference less than 3 h for RIR and 3 h and 15 min for WL or in the other direction (i.e., an increase of the oviposition time) were attributed to hens laying floor eggs. Illustrations and more details are in a previous article and its appendix [18]. However, when a hen did not lay in a nest for more than four days, it was difficult to determine her activity (laying floor eggs or pausing). Because such long periods were rare for hens with LRN ≥ 50% (≤ 0.3% of the periods without laying in a nest for both lines), only the phenotypes of such hens were used to calculate CN. Phenotypes for MOT were calculated for hens with at least 10 entries for nest visits with oviposition. For NAL, hens with LRN < 50% were assigned NAL = 0 (i.e., low ability to lay eggs in nests), while the rest were assigned NAL = 1.

Nest preference was characterized by two traits based on the location of the nests chosen for laying. The first was the mean distance between nests used for laying (MDN). To calculate this trait, nests were coded from 1 (first nest, on the left side of the pen) to n (last nest, on the right side of the pen). Nests that had the same horizontal position (top and bottom rows) had the same nest location. The distance between nests used for laying equaled the horizontal distance, expressed as the number of nests, between the nest chosen for laying on day D and the nest chosen for laying on day D − 1. To avoid including other behaviors, only nests with two consecutive laying that were not separated by one or more days without laying in the nest were considered. Accordingly, MDN was calculated using 94.2 and 89.5% of the nest visits with oviposition for RIR and WL, respectively. The other trait was the percentage of nests used for laying (PNL):

2 $${\text{PNL}}=\frac{\text{Number of different nests chosen for laying} }{\text{Total number of nests in the pen}} \times 100.$$

Unlike MDN, PNL differentiated top and bottom nests that had the same horizontal position. PNL depended strongly on the number of eggs that were laid in the nests. To correct for this effect, PNL was not calculated for hens that laid less than 100 eggs because they could not lay in as many different nests.

Three durations were calculated per nest visit with oviposition. Laying duration was computed as the time from entering the nest to exiting the nest. Duration in the nest before laying was computed as the time from entering the nest to oviposition time (when accurate). Duration in the nest after laying was computed as the time from oviposition (when accurate) to exiting the nest. Based on these, three laying duration traits were analyzed: mean laying duration (MLD), mean duration in the nest before laying (MDB), and mean duration in the nest after laying (MDA). For all nest preference and laying duration traits, except for PNL, only hens with at least 10 records were analyzed.

To better understand the genetic background of laying duration traits, the recording period was also divided into ten 28-day periods and MLD, MDB, and MDA were calculated for each period. The 10th period (59–64 weeks of age) was not kept for the RIR line due to an infection in the 2018–2019 batch, which strongly influenced nesting behavior. For each trait and 28-day period, only hens with data from at least ten records for a given trait and period were analyzed.

### Statistical analyses

Traits defined across the entire recording period were normalized using the “bestNormalize” package [27] of the R software [28] to avoid overestimation of the residual variance. They were then scaled to a mean of 0 and variance of 1 to facilitate model convergence (except the binary trait NAL). Variance components were estimated using the restricted maximum-likelihood method applied to a nine-trait animal model, separately for each line, with the hen’s hatch date as fixed. Known pedigrees of seven generations for RIR (2705 birds) and five generations for WL (2323 birds) were used to determine genetic relationships. Variance components for the 28-day laying duration traits were estimated using the same animal model as that used for the entire recording period but separately for each 28-day period, using three-trait models. For this model only, traits were not normalized and scaled before analyses because they displayed a normal distribution.

Phenotypic correlations between traits were calculated using the following equation [29]:

3 $${r}_{p}={h}_{i}{h}_{j}{r}_{a}+{e}_{i}{e}_{j}{r}_{e},$$

where ${r}_{p}$, ${r}_{a}$, and ${r}_{e}$ are the phenotypic, additive genetic, and residual correlations, respectively, between traits $i$ and $j$; $h$ is the square root of the heritability of traits $i$ and $j$; and $e$ is the square root of the proportion of residual variance for traits $i$ and $j$.

Least-square means of the laying duration traits for each 28-day period were estimated using a repeatability animal model that included the fixed effects of the hen’s hatch date and of the 28-day period, as well as the random permanent environmental effect of the hen. For a given trait and 28-day period, the least-square mean was the deviation from the last 28-day period (i.e., periods 9 and 10 for RIR and WL, respectively), which was set to 0.

All models were run using the BLUPF90 family of programs [30]. Variance components and least-square means were estimated using REMLF90. Then, a single iteration of AIREMLF90 was used to estimate standard errors of the genetic parameters.

Differences between estimates of variance components and least square means for the same trait across the 28-day periods obtained from, respectively, the three-trait and repeatability models were evaluated for significance using a two-tailed z-test. Periods were considered significantly different when the observed z-value exceeded 2.58 (α = 0.01).

## Results

### Summary statistics for nest-related traits

Respective trait means for RIR and WL were 91.80 and 86.53% for LRN, respectively, 3.98 and 8.26 for CN, and 2 h and 28 min and 3 h and 39 min after turning on the lights for MOT (Table 2). On average, hens entered the nests 1 h and 59 min and 3 h and 10 min after turning on the lights for RIR and WL, respectively (see Additional file 1: Table S1). NAL exceeded 85% for both lines. Phenotypic variance in nest preference traits was observed for both lines, with a standard deviation of approximately 3 nests for MDN and 13–16% for PNL. High exploratory behavior when choosing the laying nest was observed for some hens, with maximum values greater than 20 nests for MDN and 80% for PNL. Phenotypic variance was also observed for the laying duration traits, with standard deviations ranging from 10 to 23 min, depending on the line and trait. MLD ranged from 11 to 106 min and from 16 to 142 min for RIR and WL, respectively.

### Heritability estimates for traits related to nest preference and laying duration

Nest preference traits displayed low-to-moderate heritability estimates. The heritability estimate was moderate for MDN for RIR (0.30; Table 3) and WL (0.37; Table 4) but lower for PNL, at approximately 0.20 for both lines. Heritability estimates were high for laying duration traits, ranging from 0.54 to 0.60 (Table 3) and from 0.68 to 0.70 (Table 4) for RIR and WL, respectively.

**Table 3:** Estimates of genetic parameters and phenotypic correlations for Rhode Island Red hens

	LRN	CN	MOT	MDN	PNL	MLD	MDB	MDA	NAL
LRN	**0.13 (0.02)**	− 0.55 (0.07)	− 0.08 (0.09)	0.26 (0.10)	0.16 (0.11)	− 0.04 (0.10)	0.17 (0.10)	− 0.17 (0.10)	0.17 (0.12)
CN	− 0.37	**0.37 (0.03)**	0.78 (0.04)	0.23 (0.09)	0.17 (0.09)	0.19 (0.07)	0.19 (0.08)	0.06 (0.08)	0.19 (0.11)
MOT	− 0.10	0.52	**0.65 (0.03)**	0.29 (0.08)	0.14 (0.09)	− 0.02 (0.07)	0.11 (0.06)	− 0.12 (0.08)	0.19 (0.10)
MDN	0.04	0.06	0.00	**0.30 (0.04)**	0.85 (0.03)	0.42 (0.09)	0.43 (0.08)	0.20 (0.10)	0.17 (0.11)
PNL	0.04	0.05	− 0.04	0.72	**0.24 (0.04)**	0.46 (0.09)	0.33 (0.09)	0.39 (0.10)	0.32 (0.11)
MLD	0.04	0.13	0.05	0.16	0.12	**0.54 (0.06)**	0.72 (0.05)	0.80 (0.04)	− 0.01 (0.11)
MDB	0.08	0.15	0.06	0.28	0.19	0.70	**0.60 (0.07)**	0.19 (0.10)	0.28 (0.10)
MDA	− 0.04	0.06	0.01	0.02	0.00	0.75	0.12	**0.54 (0.06)**	− 0.24 (0.10)
NAL	NA	NA	− 0.01	− 0.03	0.05	− 0.01	− 0.02	− 0.07	**0.20 (0.03)**

Heritability estimates in bold on the diagonal, genetic correlations above the diagonal, and phenotypic correlations below the diagonal. Standard errors are in parenthesis

NA indicates that the phenotypic correlations between NAL and LRN or CN could not be calculated because all hens with LRN and CN records had NAL = 1

*LRN* laying rate in the nests, *CN* clutch number, *MOT* mean oviposition time, *MDN* mean distance between nests used for laying, *PNL* percentage of nests used for laying, *MLD* mean laying duration, *MDB* mean duration in the nest before laying, *MDA* mean duration in the nest after laying, *NAL* nest acceptance for laying

**Table 4:** Estimates of genetic parameters and phenotypic correlations for White Leghorn hens

	LRN	CN	MOT	MDN	PNL	MLD	MDB	MDA	NAL
LRN	**0.23 (0.07)**	− 0.70 (0.13)	− 0.49 (0.15)	0.06 (0.21)	0.30 (0.27)	− 0.43 (0.17)	− 0.12 (0.18)	− 0.45 (0.16)	0.26 (0.28)
CN	− 0.45	**0.52 (0.09)**	0.67 (0.09)	0.22 (0.16)	0.13 (0.25)	0.40 (0.12)	0.54 (0.11)	0.18 (0.13)	− 0.05 (0.23)
MOT	− 0.30	0.50	**0.55 (0.08)**	− 0.03 (0.16)	0.03 (0.22)	0.08 (0.13)	0.02 (0.13)	0.10 (0.13)	− 0.01 (0.20)
MDN	0.03	0.07	− 0.04	**0.37 (0.08)**	0.71 (0.21)	0.10 (0.15)	0.26 (0.14)	− 0.01 (0.15)	0.37 (0.22)
PNL	0.19	0.01	0.02	0.46	**0.18 (0.06)**	− 0.02 (0.20)	0.09 (0.21)	− 0.10 (0.20)	0.23 (0.29)
MLD	− 0.07	0.10	− 0.03	0.08	− 0.04	**0.68 (0.09)**	0.60 (0.08)	0.89 (0.03)	− 0.25 (0.20)
MDB	− 0.01	0.20	− 0.17	0.18	0.00	0.61	**0.68 (0.09)**	0.17 (0.12)	0.00 (0.20)
MDA	0.03	0.03	0.08	0.00	− 0.05	0.87	0.18	**0.70 (0.09)**	− 0.29 (0.19)
NAL	NA	NA	− 0.07	0.15	0.14	0.00	0.09	− 0.04	**0.18 (0.06)**

Heritability estimates in bold on the diagonal, genetic correlations above the diagonal, and phenotypic correlations below the diagonal. Standard errors are in parenthesis

NA indicates that the phenotypic correlations between NAL and LRN or CN could not be calculated because all hens with LRN and CN records had NAL = 1

*LRN* laying rate in the nests, *CN* clutch number, *MOT* mean oviposition time, *MDN* mean distance between nests used for laying, *PNL* percentage of nests used for laying, *MLD* mean laying duration, *MDB* mean duration in the nest before laying, *MDA* mean duration in the nest after laying, *NAL* nest acceptance for laying

### Relationships between nest preference, laying duration, and other nest-related traits

Estimates of genetic correlations between the two nest preference traits were high for both RIR (+ 0.85; Table 3) and WL (+ 0.71; Table 4). They were high for both lines for MLD with MDB and MDA (+ 0.60 to + 0.89) but lower for MDB with MDA (< + 0.20). Nest preference and laying duration traits had moderate and positive genetic correlations for RIR (+ 0.20 to + 0.46). Estimates of these genetic correlations were lower for WL (− 0.10 to + 0.10), except for the correlation between MDN and MDB (+ 0.26).

Nest preference and laying duration traits were estimated to be genetically correlated with other nest-related traits for both lines (Tables 3 and 4). Estimates of genetic correlations were low-to-moderate and positive between nest preference traits and LRN for RIR (+ 0.26 with MDN and + 0.16 with PNL) and WL (+ 0.06 with MDN and + 0.30 with PNL). Estimates of genetic correlations were low between laying duration traits and LRN for RIR, ranging from − 0.17 to + 0.17. Similarly, the genetic correlation was estimated to be low between MDB and LRN (− 0.12) for WL but stronger and negative for LRN with MLD and MDA (− 0.43 and − 0.45, respectively). Nest preference and laying duration traits had lower genetic correlation estimates with CN and MOT for RIR (− 0.12 to + 0.19), except for MDN (+ 0.23 with CN and + 0.29 with MOT). Genetic correlations were also estimated to be low between these traits for WL (− 0.03 to + 0.18), except for those for CN with MDN, MLD, and MDB (+ 0.22, + 0.40, and + 0.54, respectively). Nest preference traits were estimated to be positively genetically correlated with NAL for RIR (+ 0.17 with MDN and + 0.32 with PNL) and WL (+ 0.37 with MDN and + 0.23 with PNL). Genetic correlations between laying duration traits and NAL were estimated to be null or negative for RIR (− 0.01 with MLD and − 0.24 with MDA), except that between NAL and MDB (+ 0.28). These correlations were also estimated to be null or negative for WL (− 0.29 to 0.00).

### Laying duration traits for 28-day periods

**Fig. 1:**
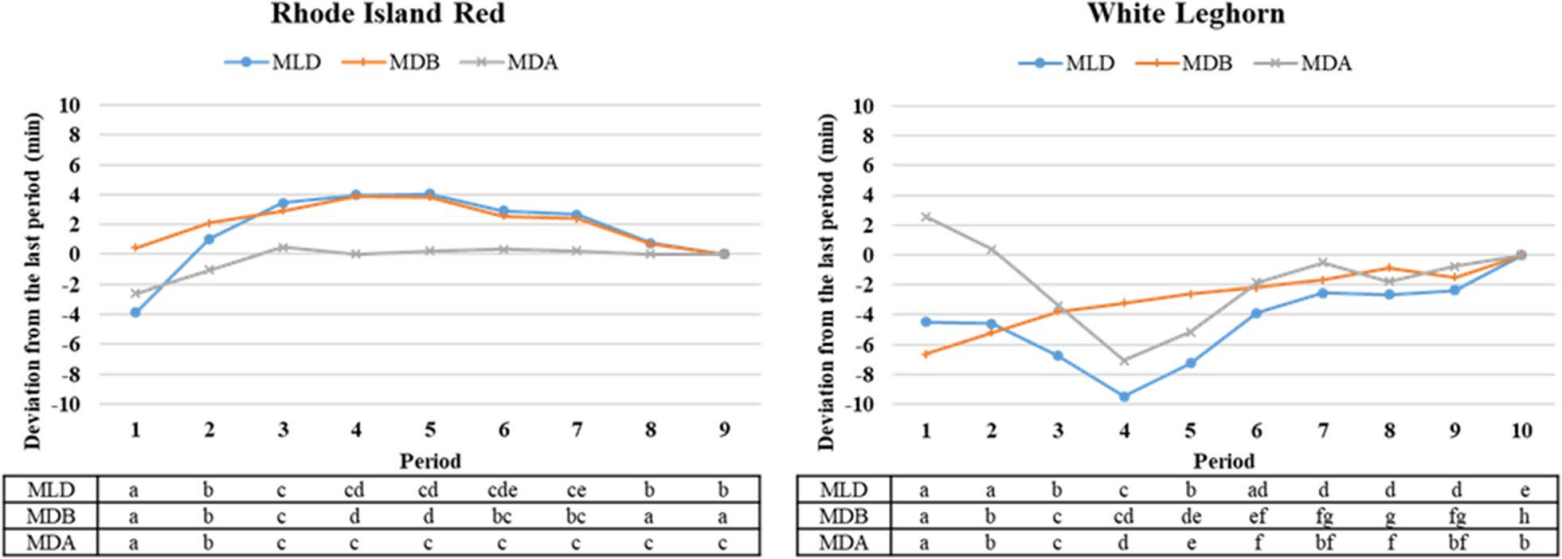
Least-square means of the laying duration traits for each line and 28-day period. *MLD* mean laying duration, *MDB* mean duration in the nest before laying, *MDA* mean duration in the nest after laying. Different letters indicate a significant difference between periods

For each line, least-square means and estimates of variance components for laying duration traits differed significantly between the 28-day periods. For RIR, least-square means of MLD increased significantly until period 3 (32–35 weeks), stabilized, and then decreased slightly during the last two periods (Fig. 1). Least-square means for MDB behaved similarly, while those for MDA did not differ significantly after period 2. For WL, least-square means for MLD and MDA decreased significantly from period 3 to 5. This decrease was mainly observed for the 2018–2019 flock when the number of eggs laid in the nest was the largest. This flock had also the higher hens per nest ratio (6.41; Table 1). However, least-square means for MDB tended to increase significantly over periods. Estimates of variance components based on the repeatability model that was used to calculate the least-square means are in Additional file 1: Table S2.

**Fig. 2:**
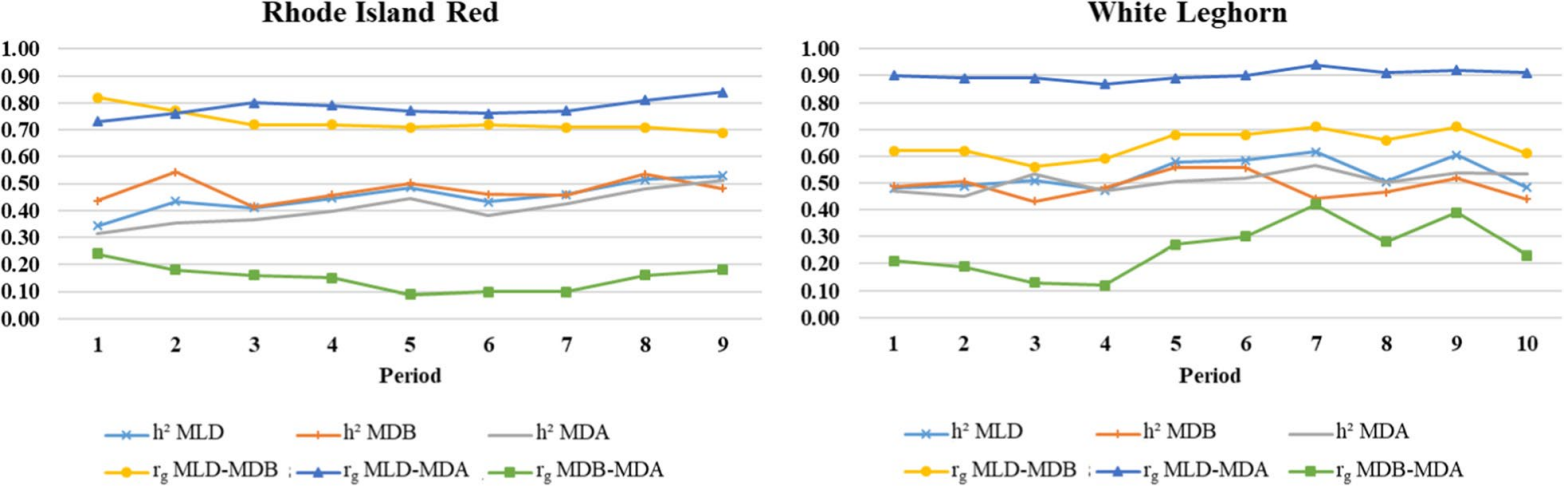
Heritability (*h*^*2*^) and genetic correlations (*r*_*g*_) of laying duration traits by line and 28-day period. *MLD* mean laying duration, *MDB* mean duration in the nest before laying, *MDA* mean duration in the nest after laying. Standard errors for *h*^*2*^ and *r*_*g*_ ranged from 0.05 to 0.12

**Fig. 3:**
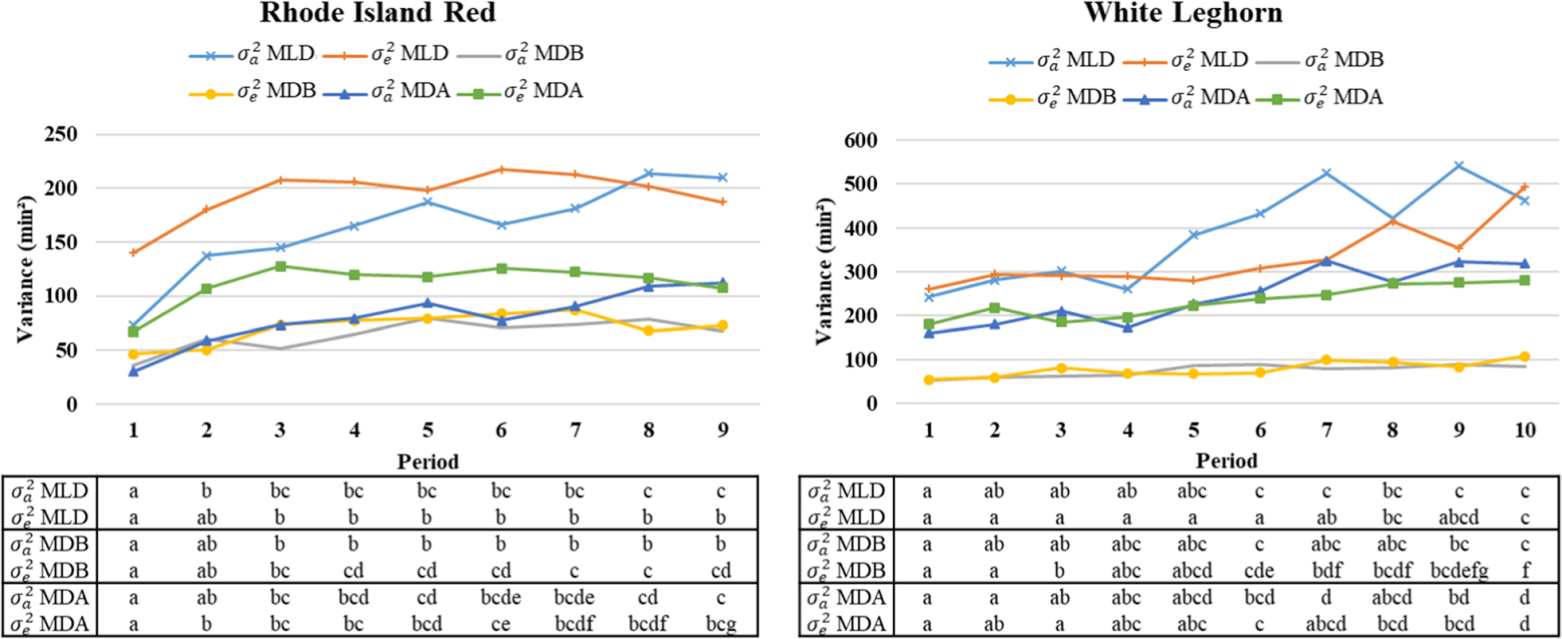
Genetic (${\sigma }_{a}^{2}$) and residual (${\sigma }_{e}^{2}$) variances of laying duration traits by line and 28-day period. *MLD* mean laying duration, *MDB* mean duration in the nest before laying, *MDA* mean duration in the nest after laying. Different letters indicate a significant difference between periods

The heritability estimates for MLD increased with age, from 0.34 to 0.53 for periods 1 to 9, respectively, for RIR but remained relatively constant for WL (mean = 0.53; Fig. 2). The heritability estimates for MDB and MDA were similar to those for MLD for both lines. Estimates of genetic correlations between the periods are in Additional file 1: Tables S3, S4, and S5. Genetic correlation estimates between MLD and MDB (mean =  + 0.73 and + 0.64 for RIR and WL, respectively; Fig. 2) and between MLD and MDA (mean =  + 0.78 and + 0.90 for RIR and WL, respectively) were relatively constant for both lines. Estimates of genetic correlations between MDB and MDA were lower, ranging from + 0.10 to + 0.24 for RIR and from + 0.12 to + 0.42 for WL. Estimates of variance components for laying duration traits tended to increase with age for both lines, except for MDB and the residual variance for MLD and MDA for RIR (Fig. 3).

## Discussion

### New phenotypes identified by using electronic nests

Differences among hens in the choice of laying nests have also been reported in the literature. For the 13 WL hens raised in a floor pen with six nests, three on each side of the pen, all but one did not choose the same nest consistently, although eight hens always used a nest on the same side of the pen for a given clutch [31]. In another study [21], the distribution of eggs laid in ten nests differed among five broiler breeder lines that were raised separately in floor pens: two of the lines preferred nests in a corner of the pen, while the other three had no preference. In two other studies of RIR raised in floor pens with three nests, hens used all three nests, but significantly preferred the nest in the corner of the pen [20, 23]. In the present study, no preference for particular nests was observed for either line, probably because the pens were larger and more nests were available, which may have made it difficult for hens to differentiate the nests [32]. The presence of roosters in floor pens may also have influenced the hens’ choice of laying site, as observed in smaller flocks of approximately 20 WL [31].

Phenotypes for MLD are similar to those reported in the literature. In one study, WL raised in eight floor pens (each with 17–18 hens) equipped with two roll-away collective nests, with different nest-floor slopes (12% or 18%), had a mean laying duration of 52–54 min, with a standard deviation of 15–16 min, depending on the slope [33]. In another study, RIR and WL hens raised in floor pens with individual electronic nests had a mean laying duration of 30 and 45 min, respectively [16]. In our study, the decrease in the least-square means for MLD and MDA from period 3 to 5 for WL (Fig. 1) could be caused by a stronger competition for accessing the nests in the 2018–2019 flock. This competition did not seem to influence MDB, which continued to gradually increase.

### Influence of genetic background on traits related to nest preference and laying duration

Heritability estimates of nest preference and laying duration traits were moderate and high, respectively, for both lines. To our knowledge, heritability estimates for traits related to nest preference for laying have not been previously reported. Nonetheless, differences in the distribution of eggs laid in nests have been observed between five broiler breeder lines, which suggests that genetic background influences nest choice [21]. Icken et al. [17] analyzed laying duration by using individual electronic nests and found that the estimates of heritability for this trait, using a repeatability model, varied greatly (0.10–0.56) depending on the laying period and flock. No heritability estimates for MDB or MDA are available in the literature. In our study, heritability estimates for nest preference and laying duration traits were generally moderate to high and similar for both lines, which suggests that the genetic background can explain some of the phenotypic variance in these traits, and likely to a similar degree for RIR and WL.

### Genetic correlations between nest-related traits tend to be favorable

Genetic correlations among nest preference traits were estimated to be strong (≥ + 0.71) for both lines, which suggests that hens that lay their eggs in nests far from each other also have a genetic predisposition to use many nests for laying. The weak genetic correlation estimates between MDB and MDA (< + 0.20) indicate that these traits are probably associated with independent behaviors. MDB is related to the “sitting” phase of pre-laying behavior, which occurs in the nest and consists of a seated posture that alternates with nest-building activities [2], while MDA may be associated with fear genes, since hens that are more afraid of their congeners or of humans might remain longer in nests after laying. Further studies are needed to better understand this behavior.

Genetic correlations between nest preference and laying duration traits were estimated to be positive for RIR (from + 0.20 to + 0.46), which suggests that selection of RIR hens with no preference for the nests used for laying may increase laying duration of their offspring. Thus, inclusion of these traits in breeding programs for RIR hens would have to take these unfavorable genetic correlations into account and use suitable weightings in the selection index.

Estimates of genetic correlations of MDN, PNL, MLD, MDB, and MDA with other nest-related traits were generally favorable, which may enable MDN and PNL to be increased and MLD, MDB, and MDA to be reduced by selection. Positive genetic correlation estimates of nest preference traits with LRN and NAL suggest that selecting hens with a good ability to choose different nests for laying would also increase egg production in nests. A recent study of broiler breeders observed that the distribution of eggs among nests depended on the genetic background and that a more uneven distribution was associated with an increase in the incidence of floor eggs [21]. Hens with no preference for particular nests can find an empty nest more easily than other hens, while hens that always lay in the same nests may have a gregarious nesting behavior and cause an uneven distribution of nests used for laying, which increases the risk of laying floor eggs [20, 21]. Based on the literature and our results, selecting for MDN and PNL, which are related to nest preference, could indirectly reduce floor eggs. However, relationships of these traits with other traits related to hen welfare, such as feather pecking or cannibalism, along with egg quality, have yet to be evaluated.

The beginning of laying period was not considered in this study (17–23 weeks of age) because differences in puberty can induce a bias on the expression of nesting behavior. For example, the estimate of the genetic correlation between LRN recorded before or after 24 weeks of age was low (0.15 and 0.42 in RIR and WL, respectively; data not shown). In addition, a recent article estimated the heritability for the percentage of floor eggs to be low at the beginning of lay (around 0.20) and then to increase [12]. Further studies are needed to better understand the relationships between the nesting behavior at the beginning of lay and later, with more records and other data such as age of puberty.

### Genetics of changes in laying duration with age

We found that the estimates of heritability for MLD increase with age for RIR (0.34–0.53) and changed little for WL (mean = 0.53). A previous study found more variable estimates of heritability for laying duration (0.10–0.56), depending on the 28-day period and flock [17]. The differences between our results and those of [17] may be explained by the fact that they used a repeatability model (versus the mean of laying duration in our study) and a smaller number of hens (206–548 hens).

For RIR, the increase in estimates of heritability for MLD with age was caused by an increase in estimates of additive genetic variance, and thus probably by the differential expression of causal genes (pleiotropic model) [34]. Similar results were observed for MDA, but not for MDB, for which estimates of heritability and variance components varied little. For WL, estimates of heritability for MLD were relatively constant with age, unlike the estimates of additive genetic and residual variances, which both increased with age, likely due to the differential expression of causal genes and the effect of age-sensitive regulatory genes, which stabilize the expression of these causal genes (epistasis model), and which increase the residual variance [34]. Similar to RIR, estimates of variance components of MLD for WL followed the same trend as those of MDA, while variance component estimates of MDB were constant with age. Thus, for both lines, MLD appears to depend more on the genetic background of MDA than MDB does, as shown by the stronger genetic correlation estimates between MLD and MDA.

## Conclusions

Moderate-to-high heritability estimates for nest preference and laying duration traits, along with the favorable genetic correlation estimates of these traits with other nest-related traits, are promising results for their inclusion in breeding programs. Selecting hens with no preference for particular nests and that spend little time in the nests used for laying can help optimize nest use, reduce nest occupation rate, and decrease incidence of floor eggs in cage-free systems. These results must be confirmed on larger populations.

## Supplementary Information

**Additional file 1: Table S1.** Summary statistics of phenotypic data for the mean time of entry (MTE) for nest visits with oviposition for the entire recording period (24–64 weeks of age). **Table S2.** Genetic (${\sigma }_{a}^{2}$), common environment (${\sigma }_{c}^{2}$), and residual (${\sigma }_{e}^{2}$) variances of laying duration traits. Variance components estimated from the model used to calculate least-square means of laying duration traits. **Table S3.** Genetic correlations between 28-day periods for mean laying duration. **Table S4.** Genetic correlations between 28-day periods for mean duration in the nest before laying. **Table S5.** Genetic correlations between 28-day periods for mean duration in the nest after laying.

## Data Availability

The data that support the findings of this study are available from Novogen, but restrictions apply to the availability of these data, which were used under license for the current study, and thus are not publicly available. However, the data can be provided upon reasonable request to Novogen.
