# Supplementary material for: Nest preference and laying duration traits to select against floor eggs in laying hens
Source: Genet Sel Evol. 2023 Jan 25;55:8. doi: 10.1186/s12711-023-00780-8 (PMC9878755; doi:10.1186/s12711-023-00780-8)
Supplement: Supplementary file 1 — Additional file 1: Table S1. Summary statistics of phenotypic data for the mean time of entry (MTE) for nest visits with oviposition for the entire recording period (24–64 weeks of age). Table S2. Genetic (${\sigma }_{a}^{2}$σa2), common environment (${\sigma }_{c}^{2}$σc2), and residual (${\sigma }_{e}^{2}$σe2) variances of laying duration traits. Variance components estimated from the model used to calculate least-square means of laying duration traits. Table S3. Genetic correlations between 28-day periods for mean laying duration. Table S4. Genetic correlations between 28-day periods for mean duration in the nest before laying. Table S5. Genetic correlations between 28-day periods for mean duration in the nest after laying. [file 12711_2023_780_MOESM1_ESM.docx]

**Table S1 Summary statistics of phenotypic data for the mean time of entry (MTE) for nest visits with oviposition for the entire recording period (24-64 weeks of age)**

|  | **Rhode Island Red** | | | | | **White Leghorn** | | | | | |
| --- | --- | --- | --- | --- | --- | --- | --- | --- | --- | --- | --- |
| **Trait** | **n** | **Mean** | **SD** | **Min** | **Max** | | **n** | **Mean** | **SD** | **Min** | **Max** |
| MTE (hh:mm) | 831 | 01:59 | 01:04 | -00:16* | 05:02 | | 920 | 03:10 | 01:02 | 00:14 | 06:10 |

n: number of hens with phenotype

SD: standard deviation

Min: minimum and Max: maximum values

*Before the lights were turned on.

**Table S2 Genetic (**$\boldsymbol{\sigma}_{\boldsymbol{a}}^{\boldsymbol{2}}$**), common environment (**$\boldsymbol{\sigma}_{\boldsymbol{c}}^{\boldsymbol{2}}$**), and residual (**$\boldsymbol{\sigma}_{\boldsymbol{e}}^{\boldsymbol{2}}$**) variances of laying duration traits**

|  | **Rhode Island Red** | | | **White Leghorn** | | |
| --- | --- | --- | --- | --- | --- | --- |
| **Trait** | $\boldsymbol{\sigma}_{\boldsymbol{a}}^{\boldsymbol{2}}$ | $\boldsymbol{\sigma}_{\boldsymbol{c}}^{\boldsymbol{2}}$ | $\boldsymbol{\sigma}_{\boldsymbol{e}}^{\boldsymbol{2}}$ | $\boldsymbol{\sigma}_{\boldsymbol{a}}^{\boldsymbol{2}}$ | $\boldsymbol{\sigma}_{\boldsymbol{c}}^{\boldsymbol{2}}$ | $\boldsymbol{\sigma}_{\boldsymbol{e}}^{\boldsymbol{2}}$ |
| MLD | 139.00 | 137.90 | 82.17 | 355.20 | 181.00 | 178.30 |
| MDB | 56.64 | 44.79 | 34.22 | 73.92 | 38.73 | 44.31 |
| MDA | 69.64 | 82.23 | 41.09 | 233.80 | 115.90 | 124.80 |

The units of variance components are min². MLD: mean laying duration; MDB: mean duration in the nest before laying; MDA: mean duration in the nest after laying.

**Table S3 Genetic correlations between 28-day periods for mean laying duration**

|  | **P1** | **P2** | **P3** | **P4** | **P5** | **P6** | **P7** | **P8** | **P9** |
| --- | --- | --- | --- | --- | --- | --- | --- | --- | --- |
| **P1** |  | 0.90  (0.05) | 0.86  (0.07) | 0.77  (0.09) | 0.77  (0.09) | 0.76  (0.10) | 0.76  (0.10) | 0.72  (0.11) | 0.69  (0.10) |
| **P2** | 0.97  (0.02) |  | 0.97  (0.02) | 0.93  (0.03) | 0.92  (0.04) | 0.90  (0.05) | 0.88  (0.06) | 0.87  (0.06) | 0.85  (0.07) |
| **P3** | 0.93  (0.04) | 0.98  (0.01) |  | 0.98  (0.01) | 0.96  (0.02) | 0.95  (0.03) | 0.94  (0.04) | 0.92  (0.04) | 0.90  (0.05) |
| **P4** | 0.96  (0.04) | 0.97  (0.02) | 0.98  (0.01) |  | 0.99  (0.01) | 0.98  (0.01) | 0.97  (0.02) | 0.95  (0.03) | 0.94  (0.04) |
| **P5** | 0.85  (0.06) | 0.96  (0.03) | 0.97  (0.02) | 0.98  (0.01) |  | 0.99  (0.01) | 0.98  (0.01) | 0.96  (0.02) | 0.95  (0.03) |
| **P6** | 0.83  (0.06) | 0.94  (0.03) | 0.97  (0.02) | 0.97  (0.02) | 0.98  (0.01) |  | 0.97  (0.01) | 0.98  (0.02) | 0.97  (0.02) |
| **P7** | 0.86  (0.06) | 0.94  (0.04) | 0.96  (0.03) | 0.96  (0.02) | 0.97  (0.02) | 0.98  (0.01) |  | 0.99  (0.01) | 0.98  (0.01) |
| **P8** | 0.86  (0.06) | 0.94  (0.04) | 0.94  (0.04) | 0.96  (0.03) | 0.96  (0.02) | 0.97  (0.02 | 0.99  (0.01) |  | 0.99  (0.01) |
| **P9** | 0.83  (0.07) | 0.90  (0.05) | 0.93  (0.04) | 0.94  (0.04) | 0.95  (0.03) | 0.97  (0.02) | 0.99  (0.01) | 0.99  (0.01) |  |
| **P10** | 0.77  (0.08) | 0.83  (0.07) | 0.87  (0.06) | 0.92  (0.04) | 0.93  (0.03) | 0.94  (0.03) | 0.97  (0.02) | 0.98  (0.02) | 0.99  (0.01) |

Rhode Island Red line above the diagonal and White Leghorn line below the diagonal. Standard errors are in parenthesis.

**Table S4 Genetic correlations between 28-day periods for mean duration in the nest before laying**

|  | **P1** | **P2** | **P3** | **P4** | **P5** | **P6** | **P7** | **P8** | **P9** |
| --- | --- | --- | --- | --- | --- | --- | --- | --- | --- |
| **P1** |  | 0.92  (0.03) | 0.87  (0.04) | 0.82  (0.05) | 0.75  (0.06) | 0.69  (0.06) | 0.68  (0.07) | 0.64  (0.07) | 0.60  (0.08) |
| **P2** | 0.96  (0.02) |  | 0.96  (0.01) | 0.92  (0.03) | 0.86  (0.04) | 0.80  (0.05) | 0.77  (0.05) | 0.76  (0.06) | 0.70  (0.07) |
| **P3** | 0.97  (0.01) | 0.98  (0.01) |  | 0.98  (0.01) | 0.95  (0.02) | 0.90  (0.03) | 0.85  (0.04) | 0.85  (0.04) | 0.79  (0.05) |
| **P4** | 0.95  (0.02) | 0.97  (0.01) | 0.98  (0.01) |  | 0.98  (0.01) | 0.96  (0.01) | 0.93  (0.02) | 0.92  (0.03) | 0.86  (0.04) |
| **P5** | 0.92  (0.03) | 0.94  (0.02) | 0.95  (0.02) | 0.99  (0.01) |  | 0.99  (0.01) | 0.97  (0.01) | 0.94  (0.02) | 0.88  (0.03) |
| **P6** | 0.91  (0.03) | 0.93  (0.03) | 0.94  (0.02) | 0.98  (0.01) | 0.98  (0.01) |  | 0.99  (0.01) | 0.97  (0.01) | 0.95  (0.02) |
| **P7** | 0.92  (0.03) | 0.91  (0.03) | 0.96  (0.02) | 0.98  (0.01) | 0.98  (0.01) | 0.98  (0.01) |  | 0.99  (0.01) | 0.97  (0.01) |
| **P8** | 0.89  (0.04) | 0.90  (0.03) | 0.93  (0.02) | 0.97  (0.01) | 0.98  (0.01) | 0.97  (0.01) | 0.98  (0.01) |  | 0.99  (0.01) |
| **P9** | 0.85  (0.04) | 0.86  (0.04) | 0.91  (0.03) | 0.96  (0.02) | 0.96  (0.02) | 0.93  (0.02) | 0.97  (0.01) | 0.96  (0.01) |  |
| **P10** | 0.77  (0.06) | 0.79  (0.05) | 0.82  (0.04) | 0.91  (0.03) | 0.91  (0.03) | 0.86  (0.03) | 0.94  (0.02) | 0.95  (0.02) | 0.98  (0.01) |

Rhode Island Red line above the diagonal and White Leghorn line below the diagonal. Standard errors are in parenthesis.

**Table S5 Genetic correlations between 28-day periods for mean duration in the nest after laying**

|  | **P1** | **P2** | **P3** | **P4** | **P5** | **P6** | **P7** | **P8** | **P9** |
| --- | --- | --- | --- | --- | --- | --- | --- | --- | --- |
| **P1** |  | 0.96  (0.01) | 0.95  (0.01) | 0.84  (0.04) | 0.88  (0.03) | 0.84  (0.04) | 0.80  (0.05) | 0.81  (0.04) | 0.78  (0.05) |
| **P2** | 0.97  (0.01) |  | 0.98  (0.01) | 0.94  (0.02) | 0.95  (0.02) | 0.92  (0.02) | 0.86  (0.03) | 0.88  (0.03) | 0.86  (0.04) |
| **P3** | 0.93  (0.03) | 0.97  (0.01) |  | 0.98  (0.01) | 0.98  (0.01) | 0.97  (0.01) | 0.92  (0.02) | 0.93  (0.02) | 0.90  (0.03) |
| **P4** | 0.95  (0.02) | 0.98  (0.01) | 0.99  (0.01) |  | 0.99  (0.01) | 0.98  (0.01) | 0.96  (0.01) | 0.95  (0.02) | 0.93  (0.02) |
| **P5** | 0.86  (0.04) | 0.96  (0.02) | 0.98  (0.01) | 0.99  (0.01) |  | 0.99  (0.01) | 0.97  (0.01) | 0.97  (0.01) | 0.95  (0.02) |
| **P6** | 0.86  (0.04) | 0.96  (0.02) | 0.98  (0.01) | 0.97  (0.01) | 0.98  (0.01) |  | 0.99  (0.01) | 0.99  (0.01) | 0.97  (0.01) |
| **P7** | 0.88  (0.04) | 0.96  (0.02) | 0.96  (0.02) | 0.96  (0.02) | 0.97  (0.01) | 0.99  (0.01) |  | 0.99  (0.01) | 0.97  (0.01) |
| **P8** | 0.88  (0.04) | 0.96  (0.02) | 0.96  (0.02) | 0.97  (0.01) | 0.96  (0.02) | 0.97  (0.01) | 0.98  (0.01) |  | 0.98  (0.01) |
| **P9** | 0.86  (0.04) | 0.93  (0.03) | 0.93  (0.03) | 0.95  (0.02) | 0.95  (0.02) | 0.97  (0.01) | 0.98  (0.01) | 0.99  (0.01) |  |
| **P10** | 0.79  (0.05) | 0.87  (0.04) | 0.91  (0.03) | 0.94  (0.02) | 0.96  (0.02) | 0.97  (0.01) | 0.97  (0.01) | 0.99  (0.01) | 0.98  (0.01) |

Rhode Island Red line above the diagonal and White Leghorn line below the diagonal. Standard errors are in parenthesis.
